# 3D Electron Microscopy Reveals the Structural Complexity of the Intravacuolar Membranous Network in *Cyrilia lignieresi*‐Infected Erythrocytes of the Fish *Synbranchus marmoratus*

**DOI:** 10.1111/jeu.70031

**Published:** 2025-07-20

**Authors:** Brenda Santarém Fachetti, Maíra Turiel‐Silva, Camila Wendt, Hilton Tulio Costi, Edilene Oliveira da Silva, Ana Paula Drummond Rodrigues, Wanderley de Souza, Kildare Miranda, José Antonio Picanço Diniz

**Affiliations:** ^1^ Universidade Do Estado Do Pará Belém Pará Brazil; ^2^ Instituto Evandro Chagas Laboratório Multiusuário de Biologia Celular e Ultraestrutura Belém Pará Brazil; ^3^ Universidade Do Estado Do Pará Marabá Pará Brazil; ^4^ Instituto de Biofísica Carlos Chagas Filho and Centro Nacional de Biologia Estrutural e Bioimagem Universidade Federal do Rio de Janeiro Rio de Janeiro Rio de Janeiro Brazil; ^5^ Museu Paraense Emílio Goeldi Belém Pará Brazil; ^6^ Universidade Federal do Pará Laboratório de Biologia Estrutural Belém Pará Brazil; ^7^ Instituto Nacional de Ciência e Tecnologia Em Biologia Estrutural e Bioimagem Rio de Janeiro Rio de Janeiro Brazil

**Keywords:** apicomplexa, cell ultrastructure, *Cyrilia lignieresi*, electron tomography, hemoparasite, intravacuolar membranous network, *Synbranchus marmoratus*, three‐dimensional reconstruction

## Abstract

This study employs advanced three‐dimensional electron microscopy techniques, including Transmission Electron Microscopy (TEM) tomography and freeze‐fracture imaging via scanning electron microscopy (SEM), to investigate the ultrastructural organization of *Cyrilia lignieresi*‐infected red blood cells (iRBCs) in the host fish *Synbranchus marmoratus* . The analysis focuses on the parasitophorous vacuole (PV) and reveals a highly complex intravacuolar membranous network (IVN) composed of vesicles, tubules, and interconnected membranous structures. These elements exhibit considerable diversity in size, morphology, and electron density, suggesting dynamic functional roles in the parasite–host interaction. The electron tomography and three‐dimensional reconstructions data provide unprecedented insights into the spatial organization and potential functional significance of these membranous systems. These findings not only enhance our understanding of the cellular adaptations of *C. lignieresi* but also contribute to a broader knowledge of apicomplexan parasitism and host–pathogen interactions.

## 1 Introduction

The Haemogregarinidae family (Apicomplexa: Adeleorina) comprises a diverse group of apicomplexan protozoa that infect a wide range of vertebrate hosts, including fish (Davies and Johnston 1976; Mamedova and Karanis 2025).

The genera *Cyrilia* (Lainson 1981), *Desseria* (Siddall 1995), and *Haemogregarina* (Danilewsky, 1885), all within the family Haemogregarinidae, differ primarily in their life cycle stages and host specificity. All utilize leeches as definitive hosts, where gametogenesis, zygote formation, and sporogony take place (Barta 2000; Siddall 1995). However, only *Cyrilia* and *Haemogregarina* undergo merogony within fish erythrocytes, whereas *Desseria* lacks this stage entirely (Davies 1995; Davies et al. 2004). Additionally, *Cyrilia* exhibits specificity for erythrocytes of freshwater fish (Lainson 1981; Mamedova and Karanis 2025), whereas *Haemogregarina* can be found in both freshwater and marine fish. In contrast, *Desseria*, although also present in marine fish, does not invade erythrocytes at any stage of its life cycle.

Developmental processes within the leech vector further distinguish these genera. In *Haemogregarina*, oocysts typically contain eight or more naked sporozoites, and meronts, appearing either rounded or worm‐like, produce 2–36 merozoites within fish blood cells. *Cyrilia* is characterized by vermiform meronts in erythrocytes and acystic sporogony in the intestinal epithelial cells of glossiphoniid leeches, generating 20–30 sporozoites from a single germinal center (Mamedova and Karanis 2025). Similarly, *Desseria* exhibits a cystic sporogony within piscicolid leeches but lacks erythrocytic merogony in the vertebrate host. Its microgametogenesis results in the production of four aflagellate microgametes (Lainson 1981; Mamedova and Karanis 2025).

To date, four species of *Cyrilia* have been described: *Cyrilia uncinata* in marine fishes of the genus *Lycodes* (Khan 1978; WoRMS 2024); *Cyrilia nili* in African freshwater fishes such as Nile tilapia ( *Tilapia nilotica* ) and African catfish ( *Clarias lazera* ), transmitted by the leech *Batracobdeloides tricarinata* (Negm‐Eldin 1999; Negm‐Eldin and Davies 1999); *Cyrilia* sp. in the Amazonian freshwater stingray *Potamotrygon wallacei* (Magro and Oliveira 2015; Oliveira et al. 2017); and *Cyrilia lignieresi* in the Amazonian freshwater fish *Synbranchus marmoratus* , transmitted by the leech *Haementeria lutzi* (Laveran 1906; Neiva and Pinto 1926; Lainson 1981).

The life cycle and ultrastructure of *C. lignieresi* have been partially characterized using light and transmission electron microscopy (TEM) (Lainson, 1981; Diniz et al. 2002). Early studies by Lainson (1981) described the parasite's life cycle in infected red blood cells (iRBCs), while Diniz et al. (2002) provided the first ultrastructural insights into the *C. lignieresi* parasite, revealing unique features such as elongated dense bodies, an invagination of the inner membrane complex (IMC), and a parasitophorous vacuole (PV) containing numerous spherical bodies.

More recently, advanced microscopy techniques, including electron tomography and focused ion beam scanning electron microscopy (FIB‐SEM), have been employed to reassess the macrogametocyte stage of *C. lignieresi*. These studies revealed additional ultrastructural details, such as tubular structures between the IMC and the parasite plasma membrane, as well as an increase in cleft‐like structures and a tubulovesicular network (TVN) in the cytoplasm of iRBCs (Turiel‐Silva et al. 2022). These findings suggest that *C. lignieresi* employs a complex system of membranous structures to interact with and manipulate its host cell.

Like other apicomplexan parasites, such as *Plasmodium* spp. and *Toxoplasma gondii*, *C. lignieresi* resides within a PV, a specialized compartment that facilitates nutrient acquisition, waste removal, and evasion of host immune responses (Adjogble et al. 2004; Bittame et al. 2015). The PV is formed during the parasite's active invasion of the host cell, a process mediated by secretory organelles such as micronemes, rhoptries, and dense granules (Bittame et al. 2015).

Within the PV, apicomplexan parasites often develop intricate membranous structures, such as the intravacuolar membranous network (IVN) observed in *T. gondii*. The IVN is thought to play a role in nutrient acquisition, protein transport, and maintenance of parasite organization within the PV, potentially influencing virulence (De Souza and Attias 2015; Magno et al. 2005; Mercier, Howe, et al. 1998). However, the precise functions of these structures remain poorly understood.

Given the unique characteristics of *C. lignieresi's* PV and its potential role in host–parasite interactions, we conducted a comprehensive ultrastructural analysis of *C. lignieresi*‐infected erythrocytes using advanced three‐dimensional electron microscopy (3D‐EM) techniques. Using TEM and SEM, including tomographic and freeze‐fracture imaging, we aimed to reconstruct and analyze the IVN and other structures within the PV. This study provides unprecedented insights into the spatial organization and potential functional significance of these membranous systems, shedding light on the mechanisms underlying *C. lignieresi's* survival and interaction with its host. Our findings contribute to a broader understanding of apicomplexan parasitism and the evolutionary adaptations of hemogregarines.

## 2 Materials and Methods

### 2.1 Blood Samples for Analysis in Electron Tomography and TEM

For this study, blood samples used for electron tomography were obtained from the archives of the Electron Microscopy Laboratory at the Evandro Chagas Institute, previously processed by Diniz et al. (2002). The blood samples from *S. marmoratus* (Osteichthyes: Synbranchidae) infected with *C. lignieresi* were routinely processed for electron microscopy (Diniz et al. 2002). ≅70 nm thin sections were stained with 5% uranyl acetate (40 min) and lead citrate (5 min) at room temperature (≅22°C), and subsequently examined using a 120 kV TEM (Tecnai Spirit, Thermo‐Fisher, Eindhoven).

### 2.2 Electron Tomography, 3D Reconstruction, and Volume Analysis

Serial ribbons of ultrathin sections, 200 nm thick, were collected on Formvar‐coated slot copper grids. Each ribbon contained an average of nine sequential sections, used for acquiring serial electron tomograms. Samples were stained with 5% uranyl acetate (40 min) and lead citrate (5 min). Later, grids were incubated with 10 nm colloidal gold on both sides for 5 min and washed in distilled water. Sections were observed in a 200 kV FEG Tecnai T20 transmission electron microscope (Tecnai T20, FEI Company, Eindhoven) equipped with a 4 k CCD camera (Eagle, FEI Company, Eindhoven). Tilt series were acquired using Xplore 3D (FEI Company). Tomograms (4096 × 4096 square pixels) were acquired between −65° and +65° with an angular increment of 1°. Of the 17 specimens observed, seven were recorded with an average of nine serial tomograms each.

For 3D reconstruction, 17 specimens in trophozoite, merozoite, and gamont stages were analyzed. The IMOD software package (Kremer et al. 1996) was used for alignment, image segmentation, 3D data analysis, and visualization. Tomogram series were aligned using the “build tomogram” workflow in Etomo. Fiducial markers were employed to improve alignment accuracy, and tomograms were generated through weighted back projection.

Cellular structures were manually segmented and rendered in 3D using 3dmod. Volume measurements of the structures of interest were extracted from the 3D models. These measurements, expressed in nm^3^, were obtained with the imodinfo plugin, which calculated the volume of the IVNs and the percentage of the total volume they occupy within the PV.

The plugin calculates the volume of segmented structures by multiplying the area of each contour by the section thickness (defined by the pixel size and *Z* scale), summing these values across all contours (Kremer et al. 1996).

The Adobe Photoshop program, version 2025, was used for the artificial coloring of the images.

### 2.3 Freeze‐Fracture Assays

For freeze‐fracture assays, 10 specimens of the fish *S. marmoratus* were captured in the municipality of Capanema, Pará, Brazilian Amazon (S 01°11′37.6″ W 047°10′01.4″), following authorization from the Chico Mendes Institute for Biodiversity Conservation (ICMBio) No. 66117‐8 and the Animal Use Ethics Committee of the Federal University of Pará No. 1622300120. Only two specimens presented mild infection on iRBCs. Infected blood samples were prepared according to the protocol of Fukudome and Tanaka (1986) with adaptations.

Samples were fixed in a solution containing 2.5% glutaraldehyde and 4% formaldehyde in 0.1 M sodium cacodylate buffer (pH 7.2) for a minimum of 2 h at room temperature. They were then carefully washed in 0.1 M sodium cacodylate buffer (pH 7.2) and postfixed in 1% OsO_4_ with the addition of 0.8% potassium ferrocyanide and 5 mM CaCl_2_ in 0.1 M cacodylate buffer (pH 7.2) for 60 min. The samples were washed three times for 10 min each in distilled water (H_2_O_d_), followed by treatment with 0.5% thiocarbohydrazide in H_2_O_d_ for 10 min, then washed again three times in H_2_O_d_ (10 min each), and finally, fixed in 1% OsO_4_ in H_2_O_d_ for 10 min at room temperature.

After the initial treatment, the samples were incubated in ECM gel (Extracellular Matrix Gel) (Sigma, USA) for 30 min at 4°C and centrifuged at 15 *g*. After removing the excess gel, the pellet was treated with a solution containing 2.5% glutaraldehyde for 12 h using a variable angle rotor model AG (FinePCR, Republic of Korea). Subsequently, the samples were post‐fixed in 1% OsO_4_ in H_2_O_d_ for 30 min at room temperature.

Next, the pellet was dehydrated in a graded ethanol series (30%, 50%, 70%, 90%, and three times in 100%), then embedded in parafilm containing ethanol, frozen in liquid nitrogen, and manually freeze‐fractured. The fracturing process involved placing the pellet on a steel plate cooled in liquid nitrogen and fracturing it using a steel blade. The samples were then transferred to chilled liquid ethanol.

For the final drying, the samples in ethanol were transferred to a critical point drying apparatus, model Leica EM CPD300 (Leica, Germany), undergoing 12 exchanges with liquid CO_2_, followed by heating, pressurization, and automatic release of CO_2_ according to the manufacturer's instructions. After complete drying, the samples were mounted on aluminum stubs, affixed with double‐sided carbon tape, and coated with a 20‐nm‐thick layer of gold. Images were acquired using a field emission gun scanning electron microscope (FEG‐SEM, Tescan Mira3, Czech Republic), operating at an accelerating voltage of 5 and 15 kV, a working distance of 7.99 and 10.73 mm, under high vacuum mode, and using a secondary electron detector.

## 3 Results

**FIGURE 1:**
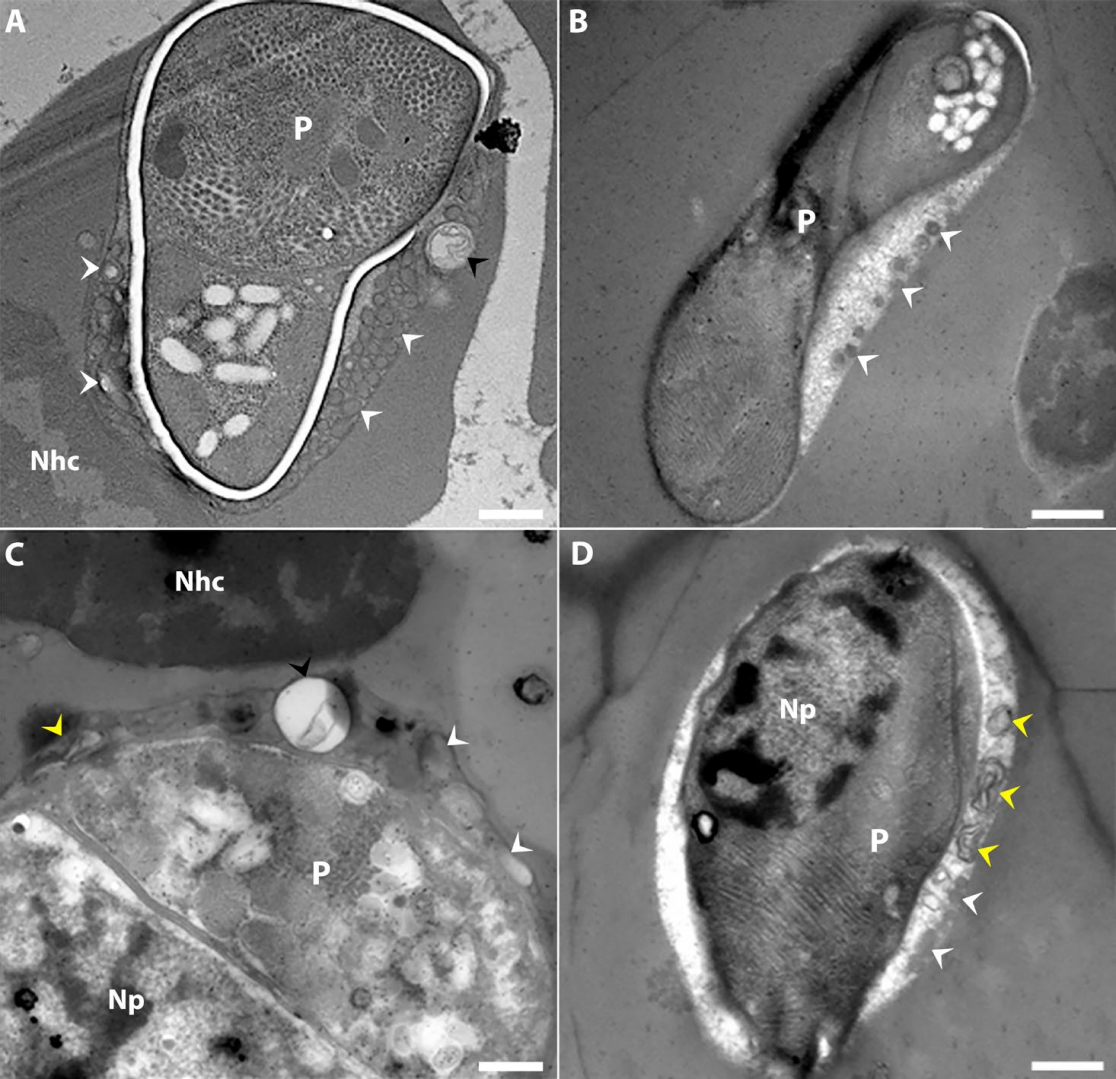
*Cyrilia lignieresi*‐iRBC in microgamont‐stage by TEM revealed the presence of membranous structures within the parasitophorous vacuole. These structures vary in number, size, morphology, and electron density (A‐D). (A) Overview of an infected erythrocyte displaying numerous electron‐dense vesicles (white arrowheads) within the PV. The host cell nucleus (Nhc) and the parasite (P) are indicated. Detail of membranous structures located asymmetrically on one side of the parasite (P; white arrowheads). (C–D) Presence of multilamellar‐like membranous structures (yellow arrowheads) and electron‐dense vesicles (white arrowheads) within the PV. In some specimens, the multilamellar‐like vesicles contained electron‐lucent material and exhibited a rounded shape (A, C; black arrowheads). The parasite (P), the host cell nucleus (Nhc, in C), and the parasite nucleus (Np) are indicated. Scale bar (A) and (C) 500 nm; (B) and (D) 1 μm.

TEM analysis of *C. lignieresi*‐iRBCs by microgamont stage revealed the presence of membranous structures within the PV (Figure 1, white arrowheads). These structures exhibited notable variability in number, size, morphology, and electron density.

In cross‐sections of *C. lignieresi*‐iRBCs, the membranous structures were abundant in some specimens, occupying a significant portion of the PV (Figure 1A), while in other cases, they were fewer in number and localized to one side of the PV (Figure 1B–D). In all cases, these structures displayed well‐defined membrane boundaries. Some specimens showed tightly packed membranous structures with minimal surrounding matrix (Figure 1A,C), whereas others exhibited a more dispersed arrangement within the PV matrix (Figure 1B,D).

The membranous structures showed different morphologies, including multilamellar‐like vesicles (Figure 1A–D) and numerous electron‐dense vesicles (Figure 1A). Small electron‐dense tubules were also observed (Figure 1C). In some specimens, the multilamellar‐like vesicles contained electron‐lucent material and exhibited a rounded shape (Figure 1A,C, black arrowhead), whereas in others, they appeared elongated, with visible membrane profiles within their contents (Figure 1C,D, yellow arrowhead).

Within the PV, vesicles varied in shape, appearing either rounded or elongated (Figure 1C). Some vesicles exhibited different stages of filling with particulate content that closely resembled the PV matrix (Figure 1C, white arrowhead). In certain specimens, vesicles within the parasitophorous vacuole were more numerous and predominantly rounded (Figure 1A, white arrowhead), often arranged around larger vesicles and multilamellar‐like structures containing electron‐dense material similar to that observed in the iRBC cytoplasm (Figure 1C,D). In contrast, membranous tubules were smaller and less abundant, also containing electron‐dense content similar to that seen in small vesicles (Figure 1C).

**FIGURE 2:**
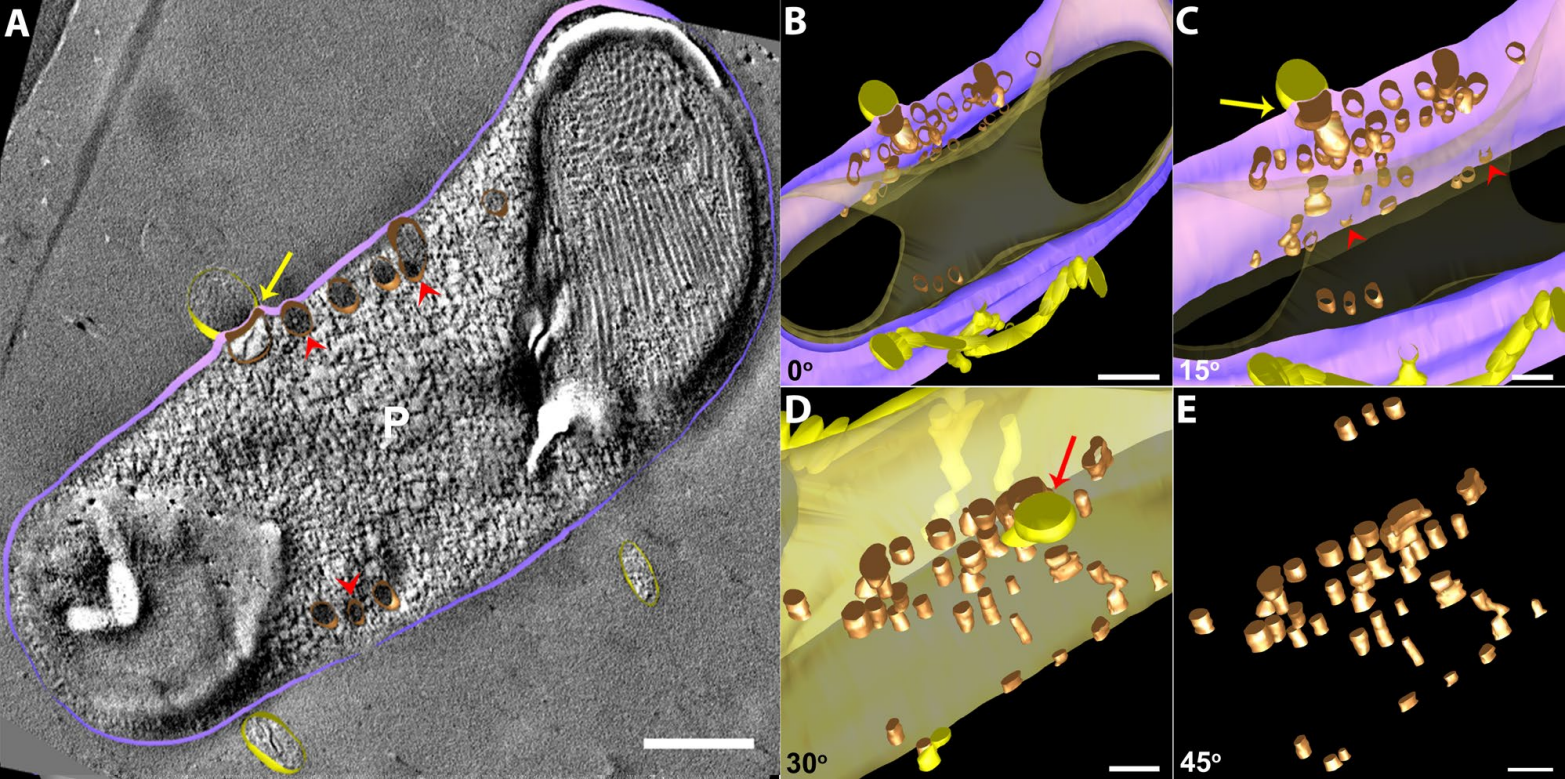
3D reconstructions of PV of the *C. lignieresi*‐iRBC in microgamont‐stage by TEM tomography revealed the IVN. In some specimens, 3D models showed that the vesicles seen in two‐dimensional images are actually unidirectional tubules distributed within the PV (A–E). (A) Note that the tubules present different content density, similar to the PV's matrix (red arrowheads), and one of the tubules very close to the TVN shows electron‐lucent content (yellow arrow). For better understanding, the images in B–E were rotated around the X axis. The contact points between the IVN and PVM (red arrowheads) are seen in (C), and (C, D) between the PVM and TVN (yellow arrow) present around the PV membrane. In E, the entire IVN. Parasite (P), brown (IVN), gray (PVM), light yellow (parasite plasma membrane), and dark yellow (TVN). Scale bar 500 nm.

Three‐dimensional reconstructions using TEM tomography on microgamont‐stage revealed that some vesicles observed in two‐dimensional images were, in fact, unidirectional tubules distributed within the PV (Figure 2A–E). In these specimens, the IVN was more abundant on one side of the PV, less compact, and surrounded by a significant amount of matrix. These structures appeared filled with particulate content (Figure 2A, red arrowheads), similar to the parasite's matrix. Conversely, the network near the TVN contained less electron‐dense material, resembling membrane profiles (Figure 2A, yellow arrow).

The volumetric analysis shows that the IVN has an average volume of 6.23 × 10^−10^ nm^3^ and occupies 7.96% of the PV's total volume (Figure 2A–C). Additionally, 3D reconstructions allowed the visualization of contact points between the IVN and the parasitophorous vacuole membrane (PVM) (Figure 2C, red arrowheads). Also note the contact points among the PVM and a TVN present around the PVM, in the iRBC cytoplasm (Figure 2C,D, yellow arrow).

**FIGURE 3:**
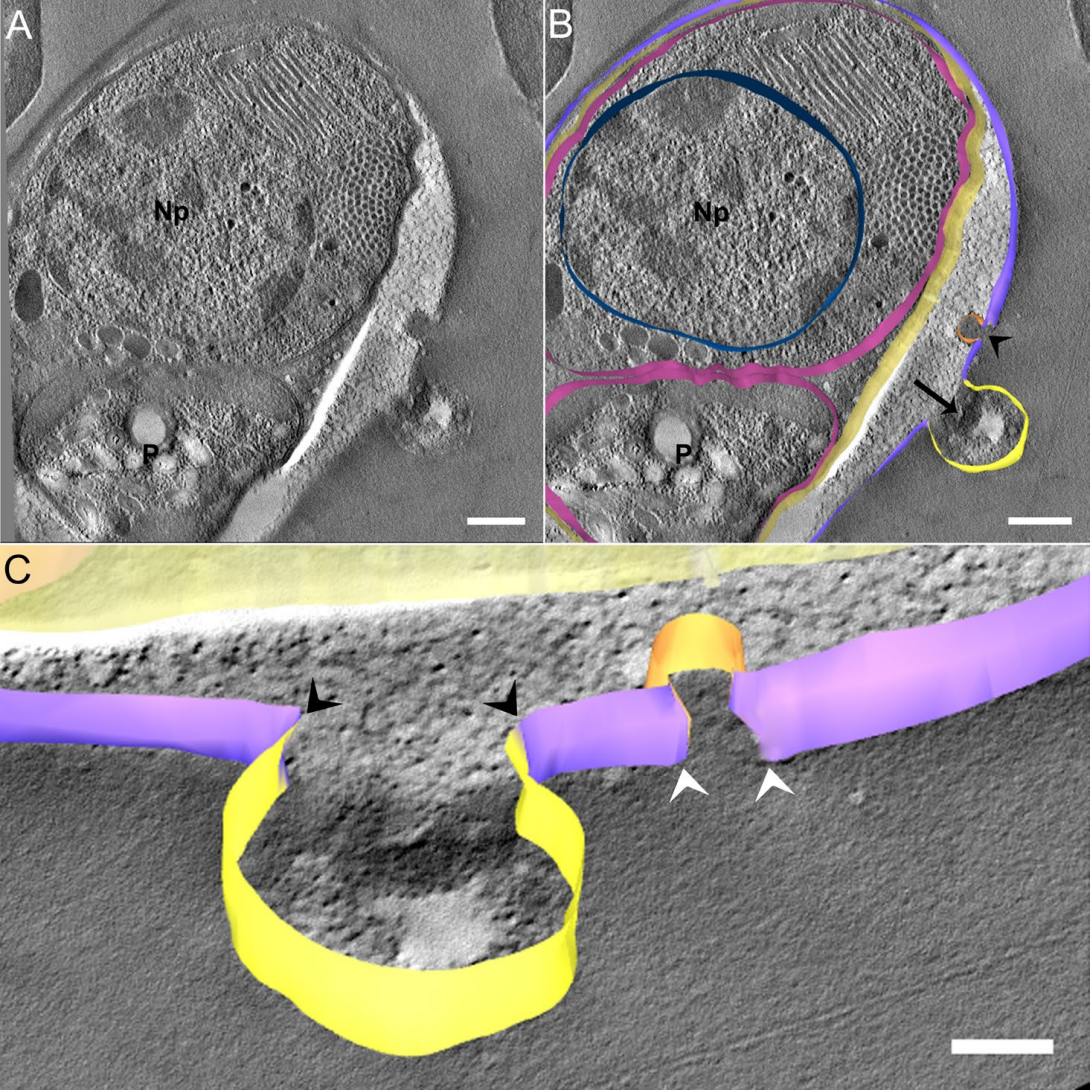
The connection points between the PVM, TVN, and the IVN were observed by serial section electron tomography and 3D reconstructions of microgamont stage. In this specimen, the tomographic images and three‐dimensional reconstructions allow visualization of what appears to be a junction between the membranes of a TVN (black arrow) and IVN (black arrowhead) with the PVM (B). In image C, we observe in more detail the opening of the PVM to the IVN (white arrowheads) and the content that appears to be shared between the structures, similar to that of the host cell cytosol. The connection between the PVM and the TVN (black arrowheads) is also observed, but the content of the sharing is heterogeneous, similar to the PV matrix. Parasite (P), (Np) Nucleus of the parasite, brown (IVN), gray (PVM), light yellow (parasite plasma membrane), and dark yellow (TVN). Scale bar (A and B) 500 nm, (C) 200 nm.

In another specimen of microgamont‐stage (Figure 3A), the IVN was smaller but appeared to fuse with the PVM (Figure 3B, black arrowhead). The IVN appears to contain electrodense material similar to that found in the host cell cytosol. Additionally, a possible membrane junction was observed between the TVN and the PVM (Figure 3B, black arrow), with heterogeneous material in the TVN resembling the PV matrix.

**FIGURE 4:**
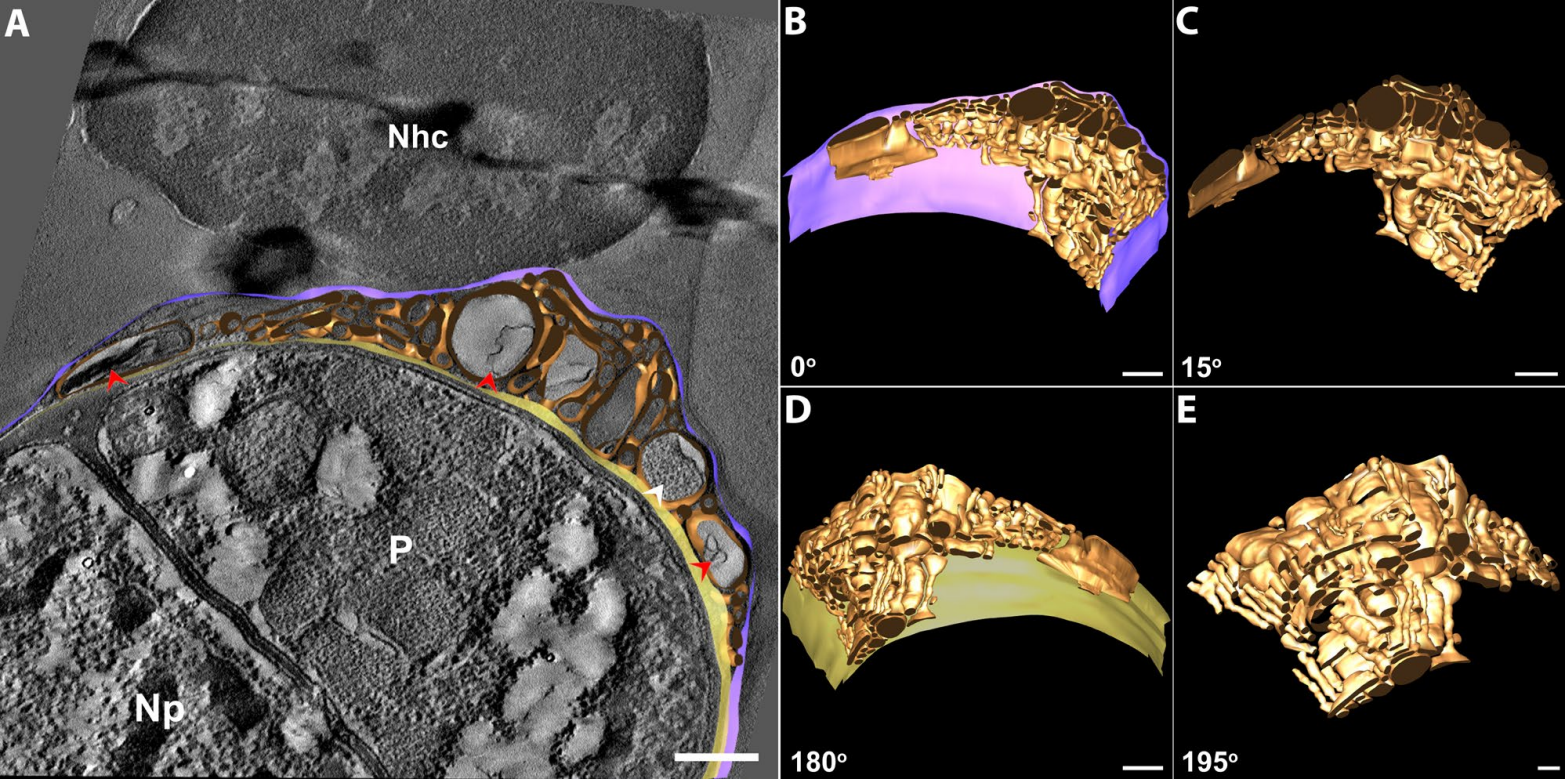
TEM tomography demonstrated the complex IVN on microgamont stage. In some specimens, 3D models showed that the membranous structures are interconnected (A–E). (A) Observe the different morphology and content of the IVN. The tubules present are sometimes partially filled (black arrowhead) or with membrane profile (yellow arrowheads). For better understanding, the images in B–E have been rotated around the X axis. The vesicles seen in two‐dimensional images are actually unidirectional tubules distributed within the PV (B–E). Parasite (P), Np (Nucleus of the parasite), Nhc (Nucleus host cell), brown (IVN), gray (PVM), and light yellow (parasite plasma membrane). Scale bar 500 nm.

In other specimens of microgamont‐stage, 3D reconstructions showed that the membranous structures are interconnected as an IVN (Figure 4A–E, Movie S1). The IVN consists of interconnected tubules with varying diameters and electron densities (Figure 4A–E). Note the proximity of these structures to each other and the scarce amount of surrounding matrix (Figure 4B–E). In this parasite, the IVN appears as a large area in cross‐sections, gradually decreasing in size throughout the parasite's PV (Figure 4A–C). In this specimen, the volumetric analysis shows that the IVN has an average volume of 7.75 × 10^−10^ nm^3^ and occupies 41.11% of the PV's total volume (Figure 4A–E).

Furthermore, in this network, a tubule with a medium diameter and rounded shape appears to be partially filled with a granulated content (Figure 4A, black arrowhead), similar to that found in the parasite's cytosol. Still, other tubules near the one with granular material have membranous profiles inside (Figure 4A, yellow arrowheads).

**FIGURE 5:**
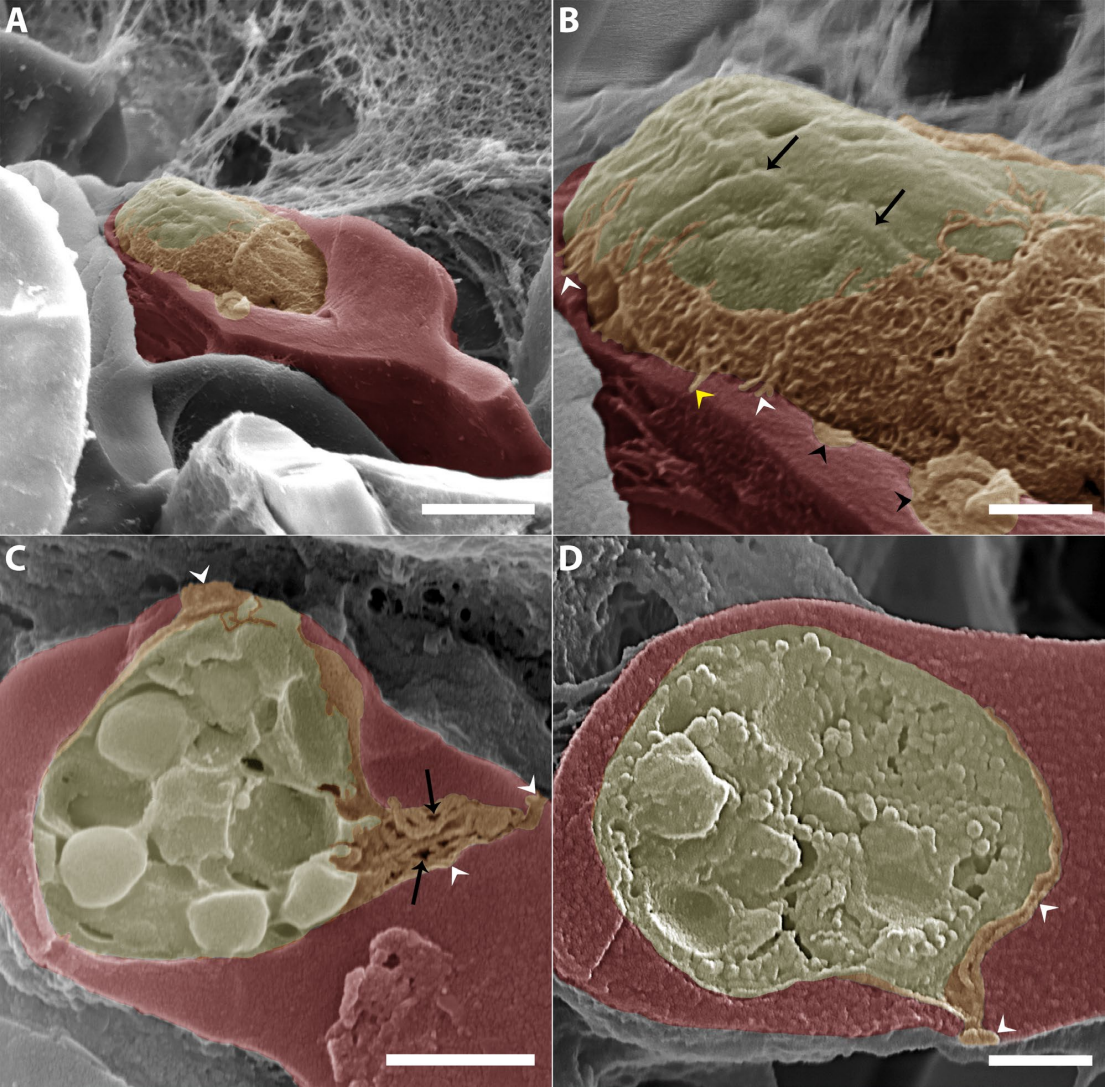
The IVN in freeze‐fractures of *C. lignieresi*‐iRBCs analyzed by SEM. In trophozoites, observe within the PV, a complex network of tubules of different diameters (A–B), including tubules extending from the IVN to the iRBC cytoplasm (B, white arrowheads). A small tubule appears to be directed toward the iRBC PM (B, yellow arrowhead). Vesicular bodies of different sizes are observed in the cytoplasm of the host cell and appear to be secreted by the parasite (B, black arrowheads). In other specimens in the young stage, seen in transverse freeze‐fracture, the IVN appears to reach the external environment of the iRBC (C–D, white arrowheads). Red (iRBC), and brown (IVN). Scale bar: (A) 2 μm; (B) 500 nm; (C) 1 μm; (D) 500 nm.

SEM of freeze‐fractured *C. lignieresi*‐iRBCs in trophozoite stage revealed a complex network of tubules within the PV, with varying diameters (Figure 5A,B). Tubules extending from the IVN to the iRBC cytoplasm were observed (Figure 5B, white arrowheads), including a small tubule directed toward the host cell plasma membrane (Figure 5B, yellow arrowhead). Vesicular bodies of different sizes, probably secreted by the parasite, were also observed in the host cell cytoplasm (Figure 5B, black arrowheads).

In transverse freeze‐fracture in the young stage, the IVN appeared to extend to the external environment of the iRBC (Figure 5C,D, white arrowheads). The network exhibited empty spaces (Figure 5C, black arrows), supporting the hypothesis that the IVN contains different levels of filling material.

**FIGURE 6:**
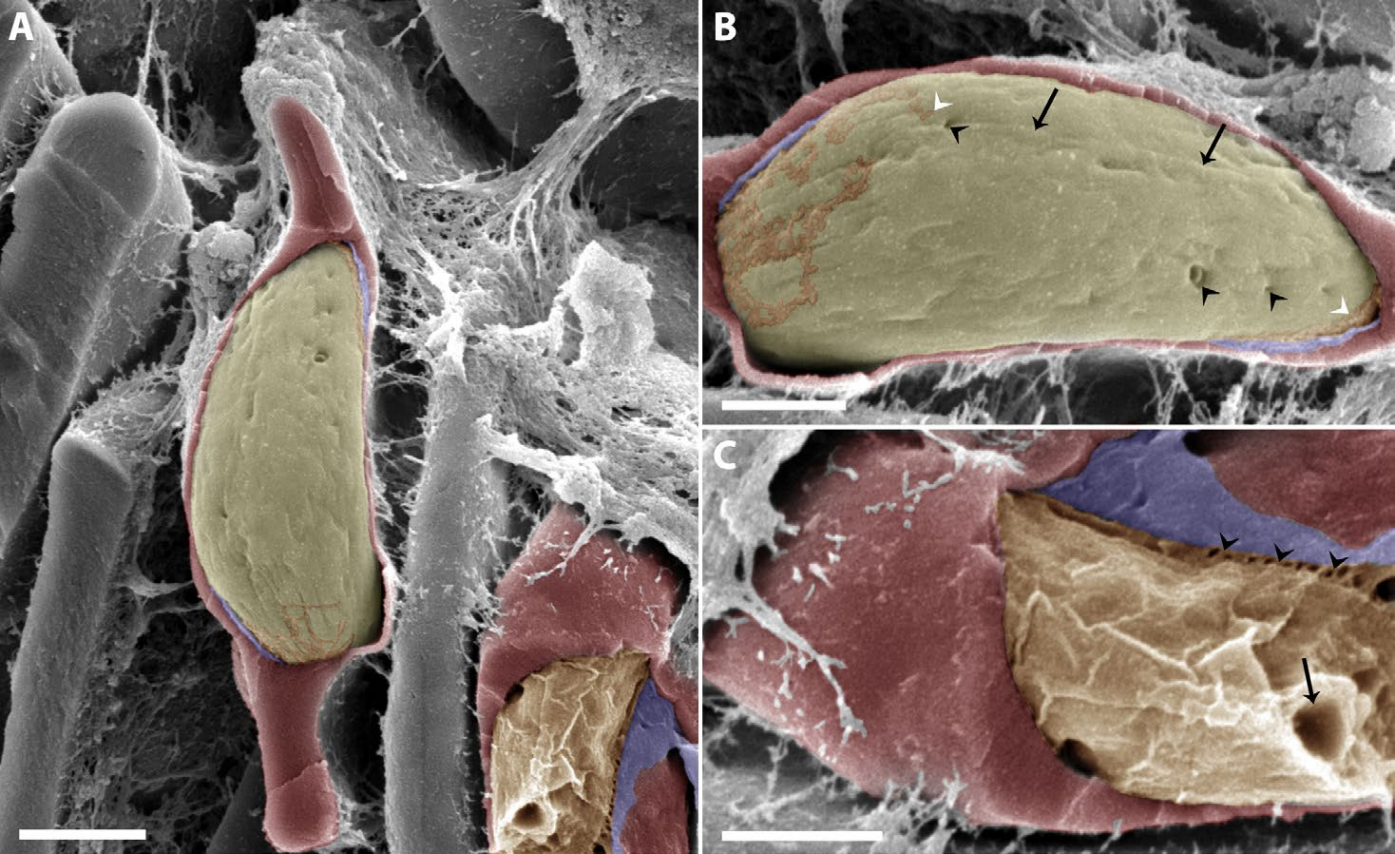
*C. lignieresi*‐iRBC parasite membrane on trophozoite stage, detailed by freeze‐fracture analyzed by SEM. The *C. lignieresi*‐iRBC presents openings in the PMs (A; B, black arrowheads), close to the IVN (A; B, white arrowheads, brown). Also, it was observed the presence of impression on the PM (A–B, black arrows). In another specimen (C), the IVN is observed as a large coat covering the parasite's body, as well as an orifice projected as if it were the interruption of a large fractured tubule (A; C, black arrow). Also, it was seen the presence of small tubules heading toward the PVM (black arrowheads) and the iRBC cytoplasm (white arrowhead) can be seen. Red (iRBC), Brown (IVN). Scale bar: 1 μm.

The images obtained by SEM also allowed the visualization of more detailed *C. lignieresi's* plasma membrane (PM) in trophozoite stage (Figure 6). It was observed in *C. lignieresi*‐iRBC the presence of openings in the PM's (Figure 6A,B, black arrowheads) with 50 nm (smallest) to 200 nm (largest) diameter, close to the IVN (Figure 6A,B, white arrowheads). Also, it was observed the presence of an impression on the PM, along the longitudinal axis of the parasite (Figure 6B, black arrows), finishing in a small pore of 50 nm (Figure 6B, black arrowhead).

In another specimen, the IVN is observed as a large coat covering the body of the parasite, as well as an orifice projected as if it were the interruption of a large fractured tubule (Figure 6A,C, black arrow). In these same images, the presence of small tubules heading toward the PVM (Figure 6C, black arrowheads) and the iRBC cytoplasm (Figure 6C, white arrowhead) can be seen.

**FIGURE 7:**
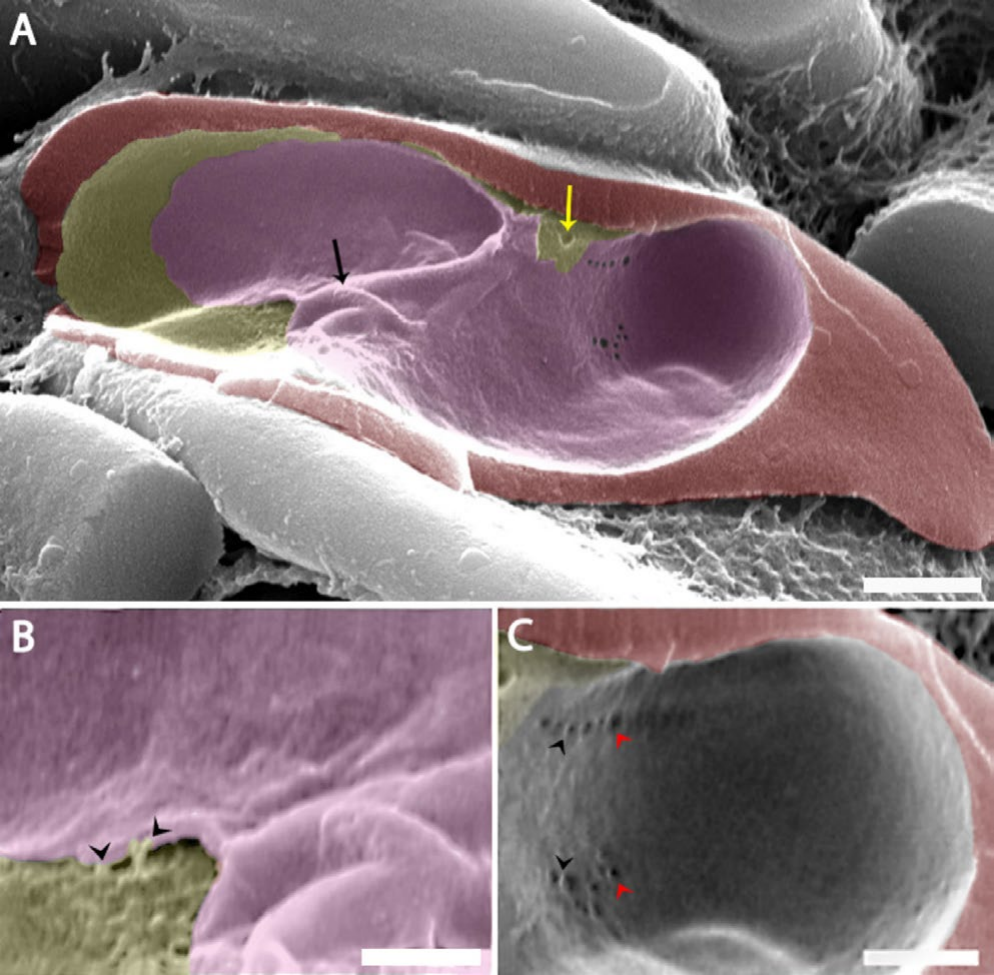
*C. lignieresi*‐iRBC IMC on macrogamont stage detailed by freeze‐fracture analyzed by SEM. The parasite was dislocated, and part of the IMC remained intact, demonstrating a slight depression on the PM (A, white arrow) and impressions of small holes in the IMC (A; C, black arrowheads). The IMC invagination is also visible (A, black arrow), along with the presence of a small tubule extending from the IMC to the PM (B, white arrowheads). Red (iRBC) and light green (IMC). Scale bar: (A) 2 μm; (B–C) 500 nm.

Freeze‐fracture SEM images revealed additional details in macrogamont stage, such as a slight depression on the PM (Figure 7A, white arrow) and impressions of small holes in the IMC (Figure 7A,C, black arrowheads). An invagination of IMC was visible (Figure 7A, black arrow), along with a small tubule extending from the IMC to the PM (Figure 7B, white arrowheads).

## 4 Discussion

In this study, we investigated *Cyrilia lignieresi*‐iRBCs to gain deeper insights into the parasite–host cell interaction, with a particular focus on the membranous structures within the PV (Diniz et al. 2002; Turiel‐Silva et al. 2022). The membranous structures within the PV exhibited significant variability in number, size, and morphology, highlighting their potential role in the dynamic interactions between the parasite and its host. The distribution of these structures varied among samples, with some parasites displaying an abundance of vesicles distributed throughout the PV, while others showed more localized structures confined to one side of the vacuole. These differences suggest potential adaptive functions or structural differentiation among parasites, possibly reflecting different stages of infection or host–parasite interactions.

TEM analysis revealed a wide range of vesicles and tubules within the PV of *C. lignieresi*‐iRBCs, which probably play a critical role in the parasite's intracellular organization. These structures varied significantly between specimens; some parasites exhibited large clusters of electron‐dense vesicles, while others displayed smaller, more dispersed structures. The membranous structures included multilamellar‐like vesicles and electron‐dense tubules, with some vesicles containing particulate or electron‐lucent material. Notably, microvillus‐like projections from the plasma membrane (PM) of iRBCs, similar to those observed in our study, have also been documented in *Haemogregarina* sp. by Baker and Lainson (1967), as well as in human cells infected with *T. gondii* (Sibley 1995; Mercier et al. 2005; Travier et al. 2008).

Three‐dimensional reconstruction using TEM tomography revealed that some of the heterogeneous vesicles observed in two‐dimensional sections, such as multilamellar, rounded, or particulate‐containing bodies, were, in fact, interconnected tubules forming a membranous network between the parasite plasma membrane and the PVM. These IVNs exhibited two distinct morphological patterns: (1) a highly branched network and (2) simplified, unidirectional arrangements. The volumetric variation of the IVN (0.96%–41.11% of the PV volume) suggests that its formation may be regulated by metabolic demands or the intracellular stage of the parasite. This behavior resembles the tubulovesicular network of *Plasmodium falciparum*, whose morphogenesis depends on sphingolipids and is linked to solute transport between the parasite and the host cell (Haldar et al. 2001; Lauer et al. 2000). However, unlike the filamentous IVN of *Toxoplasma gondii*, which is primarily associated with parasite rosette maintenance (Magno et al. 2005), the IVN in *C. lignieresi* appears to be functionally involved in metabolic exchange with the host cytoplasm.

In *T. gondii*, the IVN is composed of tubular and vesicular membranes that connect the parasites to each other and the PVM (Guevara et al. 2019; Mercier et al. 2002). These tubules can ramify and merge while maintaining a uniform diameter (De Souza and Attias 2015; Portes et al. 2023). In contrast, the IVN of *C. lignieresi* exhibits variability in tube diameter, suggesting functional differences in network organization. In *T. gondii*, the IVN forms after tachyzoite dense granules secrete GRA proteins into the PV space shortly after initial infection (Mercier et al. 2002; Travier et al. 2008). Given these findings, it is worth investigating whether a similar process occurs in *C. lignieresi*: are dense granules involved in the formation of the IVN in *C. lignieresi*? Further studies, including cytochemical and immunohistochemical analyses, as well as specific labeling of secreted proteins, are necessary to address this question. SEM provided detailed images of the PM and revealed novel features such as pores and depressions on the surface of *C. lignieresi*. These pores, ranging from 50 to 200 nm in diameter, were located near the IVN, suggesting a functional relationship between the network and the host cell's PM. The pores observed in *C. lignieresi* differ from the micropores found in other apicomplexan parasites such as *Plasmodium* spp., *Theileria* spp., and *Babesia* spp. (Yang et al. 2024). The number and distribution of pores and depressions can vary widely among apicomplexan parasites (Yang et al. 2024). For example, *Plasmodium* spp. may exhibit a greater number of pores at specific life cycle stages, while *T. gondii* may display deeper depressions (Wan et al. 2023). The function of these pores remains poorly defined, as they were only observed under SEM and not in TEM analyses. One possibility is that these pores are involved in exocytosis or endocytosis processes, possibly facilitating the secretion of the IVN described here. Further TEM studies are needed to evaluate the protein cytoarchitecture of these pores and their potential role in parasite biology.

The interaction between the IVN and host cell structures is of particular importance. In some *C. lignieresi* specimens, the IVN extended beyond the PV, potentially interacting with the external environment of the iRBC. The presence of vesicular bodies in the iRBC cytoplasm further supports the idea of secretion activities by the parasite. This complex interaction is reminiscent of other apicomplexan parasites, such as *T. gondii*, where GRA proteins (e.g., GRA2, GRA4, GRA6) are associated with the IVN, while others (e.g., GRA3, GRA5, GRA7, GRA12) are incorporated into the PVM, influencing host cell functions (Vommaro et al. 2014; Portes et al. 2023).

This pattern of minimal host cell alteration aligns with what is observed for *Hepatozoon ixoxo*, which, despite being encapsulated and exhibiting a polar cap, induces few morphological changes in host erythrocytes (Conradie et al. 2017). In contrast, *H. theileri* and *H. damiettae* cause prominent cytopathological effects such as hypertrophy, dehemoglobinization, karyolysis, and membrane protrusions supported by microtubule‐like elements (Conradie et al. 2017; Mansour 2025). In *C. lignieresi*, the preservation of erythrocyte morphology suggests a distinct immune evasion strategy, possibly mediated by the IVN acting as a specialized secretory system. This subtler manipulation of the host cell may allow the parasite to establish a stable intracellular niche while avoiding detection. Supporting this, 3D reconstructions revealed connections between the IVN, TVN‐like structures, and the parasitophorous vacuole membrane, suggesting an integrated functional network. These findings reinforce the hypothesis that *C. lignieresi* has evolved adaptive strategies that enable efficient intracellular manipulation with minimal disruption to the host cell membrane architecture.

This comparison suggests that *C. lignieresi* manipulates its host cell more subtly, primarily affecting the internal cytoarchitecture without compromising the external integrity of the erythrocyte membrane. Such a strategy may reflect an evolutionary adaptation aimed at evading the host immune response by preserving the external phenotype of the infected cell. While *H. theileri* appears to more aggressively compromise host cell integrity, possibly to facilitate nutrient access or parasite egress, *C. lignieresi* may rely on the IVN to establish a stable intracellular niche with minimal visible structural disturbance. This divergence in cell remodeling mechanisms highlights distinct evolutionary strategies among hemogregarines and raises important questions about the relationship between parasite structural organization, transmission dynamics, and host immune modulation.

This intricate approach to host cell manipulation may reflect the unique ecological context of these parasites. In the Brazilian Amazon, the prevalence of *Cyrilia* species may be related to the association between host animals and their environment, particularly the alternating marine and freshwater habitats. Amazonian fishes have developed adaptive strategies to survive cyclical flooding (Almeida‐Val et al. 1999). Building on this ecological adaptation, it is plausible that hemoparasites like *C. lignieresi* have evolved similarly, influenced by these environmental dynamics. Future studies are essential to further elucidate the developmental cycle of these hemoparasites and to investigate their potential effects on the metabolism of *Synbranchus marmoratus* , a native Amazonian fish, in response to parasitic infection.

The results presented in this study, combined with previous data on the prevalence of *Cyrilia* species in Amazonian environments, suggest a complex co‐evolution between the parasite and its host, shaped by the unique characteristics of the ecosystem. The ability of *C. lignieresi* to manipulate the host cell, combined with its flexible life cycle, indicates an adaptation to a dynamic and changing environment. Future research should focus on exploring the genetic diversity of *C. lignieresi* across different Amazonian fish populations to further elucidate its evolutionary processes and environmental interactions. Unfortunately, studies on the *Cyrilia* genus are scarce, and even fewer provide electron microscopy data to expand our understanding of the structures described here. A detailed understanding of the structure and function of the *C. lignieresi* IVN is essential to determine how the network influences the parasite's virulence and its ability to establish infection. Characterization of the IVN reveals an unexpected complexity in host–parasite interactions. Identification of the effector proteins secreted by the IVN and their targets in the host cell is crucial to elucidating the molecular mechanisms underlying cellular manipulation. Proteomics and functional genomics experiments, combined with ultrastructural analyses, will provide deeper insights into the organization and function of the IVN and its role in pathogenesis.

Despite that, the results of this study highlight the importance of a multidisciplinary approach to the study of *C. lignieresi*. The integration of cell biology techniques, electron microscopy, and phylogenetic analysis is fundamental to deciphering the complex dynamics of this parasite. Moving forward, it will be crucial to combine ecological, evolutionary, and molecular perspectives to fully comprehend the interaction between *C. lignieresi* and *S. marmoratus*.

## Conflicts of Interest

The authors declare no conflicts of interest.

## Supporting information


**Movie S1.** The IVN details of C. lignieresi‐iRBCs on microgamont stage analyzed by TEM tomography and 3d reconstruction. The IVN present in the PV of C. lignieresi‐iRBC is best visualized through virtual sections and three‐dimensional reconstruction. Note that the network in two‐dimensional planes appears to contain vesicles with different contents and morphologies. After three‐dimensional reconstruction, the vesicle structures merge and divide within an intricate and complex network.

## Data Availability

The data that support the findings of this study are available from the corresponding author upon reasonable request.
